# Effects of Non-Invasive Radiofrequency Diathermy in Pelvic Floor Disorders: A Systematic Review

**DOI:** 10.3390/medicina58030437

**Published:** 2022-03-17

**Authors:** María Dolores González-Gutiérrez, Álvaro López-Garrido, Irene Cortés-Pérez, Esteban Obrero-Gaitán, Felipe León-Morillas, Alfonso Javier Ibáñez-Vera

**Affiliations:** 1 Centro de Fisioterapia Lola González, Calle Cronista Salcedo Hierro, 8, 14001 Cordoba, Spain; lolagonzalezgutierrez@hotmail.com; 2 Clínica Cefire, Calle Blas Infante, 3, 23400 Úbeda, Spain; alg00064@red.ujaen.es; 3 Department of Health Sciences, University of Jaen, Campus las Lagunillas, 23071 Jaén, Spain; icp00011@red.ujaen.es (I.C.-P.); ajibanez@ujaen.es (A.J.I.-V.); 4 Poniente de Almería Northeast Health District, Andalusian Health Service, 04740 Roquetas de Mar, Spain; 5 Department of Physiotherapy, Catholic University of Murcia UCAM, Avenida de los Jerónimos, 30107 Murcia, Spain; fleon@ucam.edu

**Keywords:** diathermy, radiofrequency, capacitive–resistive therapy, dielectric, pelvic floor disorders, pelvic pain, pelvic floor dysfunction

## Abstract

*Background and Objectives*: In recent years, the use of radiofrequency diathermy in pelvic floor disorders has grown proportionally to the interest in this specialty. Despite the common use of this therapy among pelvic floor physiotherapists, little is known about its effects and effectiveness in pelvic floor disorders. For this reason, the aim of the present review is to assess the effects of non-invasive 300 kHz–1 MHz radiofrequency diathermy in the treatment of pelvic floor disorders. *Materials and Methods*: A literature search was performed in PubMed, Scopus and Web of Science, searching for any type of study that included pelvic floor disorder participants and an experimental group treated with non-invasive nor ablative radiofrequency diathermy. *Results*: There were a total of 578 studies after removing duplicates. The inclusion and exclusion criteria were applied, resulting in a total of 15 studies, which were methodologically assessed with PEDro and the Newcastle and Ottawa scale. *Conclusions*: Despite the low quality of most of them, the studies showed improvements in urinary incontinence, pelvic pain conditions, pelvic floor muscles strength and sexual function. These findings must be considered with caution until more randomized clinical trials are performed to solve the biases detected.

## 1. Introduction

Radiofrequency diathermy (RFD) is a non-invasive therapy that consists of the emission of high-frequency electromagnetic waves, which produce deep heat that increases the metabolism of biological tissue [1]. This process promotes tissue repair and influences pain sensitivity [2,3]. Wavelengths of 300 kHz to 1 MHz are commonly used among physiotherapists for pain management and reducing the recovery time of musculoskeletal disorders [4]. Regarding technical aspects, there exist two types of devices regarding energy transmission: capacitive–resistive and dielectric. Moreover, these devices could also be classified according to the number of poles (monopolar or bipolar), the latter including other multipolar devices that are merely a bipolar with more than one output of a pole [5]. Despite the variety of devices and according to our knowledge, there are no studies comparing the effects of different devices on the same pathological condition.

“Pelvic floor disorders” is a term that encompasses any dysfunction related with the pelvic floor: urinary incontinence, dyspareunia, chronic pelvic pain, erectile dysfunction, postpartum symptoms, pre- and postmenstrual syndrome, etc. Physiotherapy has an important role in most of the mentioned disorders, being considered the main therapeutic approach in many of them [6,7]. In this line, the use of RFD as treatment in pelvic floor disorders has grown in recent years due to its metabolic and analgesic effects, which could be helpful in many disorders. Kumaran and Watson defined the deep blood flow changes that appear with the application of RFD, which enhances blood support to soft tissues [1]. Other authors focused their research on the analgesic effects of RFD on musculoskeletal [8,9,10], neuropathic [11], myofascial [12] and post-surgery pain [13]. Nevertheless, Gold et al. [14] reviewed its efficacy in feminine rejuvenation, suggesting improvements in mild to moderate conditions related with vaginal laxity and neocollagenesis proliferation [15], despite the lack of robustness in the quality of the studies found [14]. Although its use is widely accepted, no systematic reviews have been performed to evaluate the existing evidence about the use of RFD in pelvic floor disorders. For this reason, the aim of the present review is to assess the effects of non-invasive 300 kHz–1 MHz RFD in the treatment of pelvic floor disorders.

## 2. Materials and Methods

### 2.1. Study Design

A systematic review was carried out in accordance with the Preferred Reporting Items for Systematic Reviews and Meta-Analyses (PRISMA) (updated version (2020)) guideline [16] and the Cochrane Handbook for Systematic Reviews of Interventions of Higgins and Green (version 2011) [17]. In addition, the PROSPERO register was searched and no prior systematic review of our topic of interest was identified.

### 2.2. Literature Search

Two researchers independently performed a bibliographic search on PubMed Medline, Scopus and Web of Science databases until December 2021. Additional searches were performed in previously published reviews, books, practice guidelines and gray literature (expert documents and conference proceedings). In order to follow a systematic search strategy, the PICO system was used to develop the bibliographical search chains [17]: Population (patients with pelvic floor disorders), Intervention (radiofrequency diathermy), Comparison (comparison of patients treated with radiofrequency diathermy with sham/placebo therapy or any other treatments) and Outcomes (any related with pelvic floor disorders). The keywords used in this search strategy, according to the Medical Subjects Headings (MeSH), were “radiofrequency therapy”, “hyperthermia induced”, “pelvic floor”, and “pelvic floor disorders”, with its entry terms. Boolean operators “AND”/“OR” were used. In addition, a third researcher with expertise in search strategy took part to resolve any doubts. Table 1 shows the search chains used in each of the databases.

### 2.3. Study Selection

This stage was performed by two authors that independently screened the retrieved articles of each database by title and abstract. If one author considered an article as a possible study to be included in the systematic review, it was examined in detail. A third author was consulted to solve any disputes. A study was included in the review if it met all the following inclusion criteria: (1) trials or cases studies using RFD (from 300 kHz to 1 MHz) as main treatment or part of it; (2) with a sample comprising participants diagnosed with pelvic floor disorders; (3) all studies included, almost must to have an intervention group that perform RFD.; (4) the results of the study must report data concerning pelvic floor function; and (5) studies must have been published in the last 10 years. Otherwise, studies were excluded that: (1) were duplicated; (2) exclusively analyzed outcomes related with pelvic floor rejuvenation or aesthetics; (3) did not report results related with pelvic floor health; (4) used interventional or minimally invasive radiofrequency.

### 2.4. Data Extraction

Two authors performed data extraction independently, with any discrepancy being resolved by a third researcher. Data included pelvic floor disorders, outcomes, therapy groups, sample size, participants per group, year of publishing, authors, age, sex, adverse effects registered and type of RFD application. Finally, results were also considered to assess the efficacy of the treatment.

### 2.5. Outcomes

Due to the aim of this systematic review, the main outcomes considered were perceived pain in any part of the pelvic floor, gender interference, sexual satisfaction, urinary incontinence, menopausal symptoms and pelvic floor muscles’ strength. Secondary outcomes included: erectile dysfunction, time of orgasm, allodynia, hyperalgesia and quality of life.

### 2.6. Methodological Quality Assessment

The PEDro scale was the tool used to assess the methodological quality of studies that were designed as randomized clinical trials [18]. Case series or simple trials were evaluated with the Newcastle–Ottawa Scale (NOS) [19]. The PEDro scale consists of 11 items, each counting as one point with the exception of the first one, which is related to external validity, so the scale scores from 0 to 10. A score of 9–10 indicates excellent methodological quality studies, 6–8 for good-high quality ones, 4–5 for moderate quality and under 4 in the case of low quality [18]. For assessing other non-randomized studies, the NOS allows for analysis of the quality of selection methods, comparability among groups and exposure to interventions [19]. Two researchers independently assessed the methodological quality of the studies included in the review and a third researcher was consulted to resolve the discrepancies that arose.

## 3. Results

### 3.1. Study Selection

**Figure 1:**
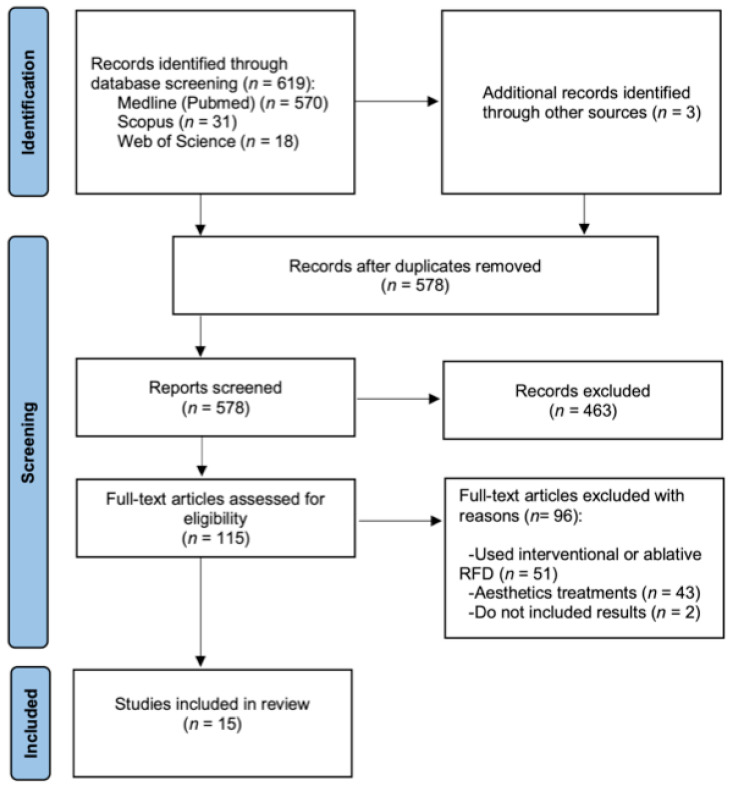
Flow diagram of the study search.

The PRISMA flow chart (Figure 1) shows the study selection process. Six-hundred-nineteen studies were found among PubMed, Scopus and Web of Science, which were added to another three found by references. From those, 44 were discarded as duplicates. The title and abstract of the remaining 578 were screened and 463 were excluded due to the selection criteria. In the end, 100 more studies were excluded after reading the whole text, obtaining 15 studies that met the inclusion criteria [20,21,22,23,24,25,26,27,28,29,30,31,32,33,34].

### 3.2. Characteristics of the Studies Included in the Review

A total of 15 studies included in the review were: 5 RCT and 10 other study designs (7 case series, 2 one-arm non-RCT and 1 prospective study). Studies included data of 635 participants with different pelvic floor disorders (sexual function disorders, stress urinary incontinence, endometriosis-related pain, chronic pelvic pain, postpartum perineal pain, Peyronie’s disease and post-menopausal sexual symptoms). In addition, a summary of the main characteristics of the studies such as author, year, disorder, sample, outcomes, intervention and results are represented in Table 2 (randomized controlled trials) and Table 3 (other study designs).

### 3.3. Methodological Quality Assessment

The randomized controlled trials that were selected obtained moderate quality on the PEDro scale: three scored 5/10 [20,27,29], one 6/10 [21] and the last 7/10 [28]. These results suggest that higher quality randomized controlled trials are necessary on this topic, as the existing studies present several limitations. Concerning other study designs, they were assessed by the Newcastle and Ottawa scale, obtaining low scores: between 0 and 3 stars [22,23,24,25,26,30,31,33,34,35]. This poor result is explained by the lack of comparison groups in most of the designs and the possible risk of selection bias due to the lack of information about participants’ identity and recruitment. Supplementary Tables S1 and S2 show the score item by item of both methodological assessment scales.

### 3.4. Synthesis of Outcomes and Questionnaires Used in Studies Included in This Review

From the 15 studies selected, 6 were related to the effects of RFD in urinary incontinence (5 in women [22,23,29,31] and 1 in prostatectomized men [34]). With this aim, a variety of tools were used to assess the urinary loss of participants (Urogenital Distress Inventory (UDI-6), International Consultation on Incontinence Questionnaire for Urinary Incontinence Short Form (ICIQ-SF UI), Female Sexual Function Index (FSFI), International Consultation on Incontinence Questionnaire for Overactive Bladder (ICIQ-OAB) and the pad test), which made it difficult to meta-analyze the effects. All studies found improvements after treatment with RFD in some of the evaluation tools used. Considering the validity of the measures, most of them were validated tools (UDI-6 [36], ICIQ-SF UI [37], FSFI [38], ICIQ-OAB [39]), although the pad test suffers from frequent variations in how the test is performed, which reduces its generalizability [40].

### 3.5. Synthesis of Main Findings

Regarding sexual function, two RCTs measured the effects of RFD in FSFI, revealing improvements of 1.8 (*p* = 0.031) [21] and 3.51 (*p* = 0.03) [28], respectively. Another case series study [26] and a non-randomized trial [22] analyzed the effects of therapy on women orgasmic dysfunction, obtaining a self-perceived reduction of 50% in time to orgasm for the first [26] and no changes for the second [22].

Postpartum perineal pain was treated in the RCT of Bretelle et al., finding no differences in perineal pain but improvements in discomfort while walking in the experimental group [20]. A case series of Dayan et al. obtained improvements in muscle maximum contraction after postpartum, not in basal muscle tone [33], using a biofeedback device for this assessment.

There were also studies focused on pain. Fernández-Cuadros et al. obtained a reduction of 3.52 points on the VAS (*p* < 0.0001) in a case series of women with pelvic pain or dyspareunia [35], while Fortún-Rabadan reduced referral pain and dysmenorrhea intensity in a case series with five women [30]. Nonetheless, an RCT evaluated the effects of RFD in 94 participants with Peyronie’s disease, obtaining a reduction of 2 points on the VAS (*p* < 0.01) during erection, although no changes in curvature nor International Index of Erectile Function were observed [27].

## 4. Discussion

The application of RFD in pelvic floor disorders reports interesting perspectives, but undoubtedly, evidence is still far from supporting its use as a main therapy. In fact, very few studies have sufficient quality and are free of bias to support the use of RFD as something more than a complementary therapy to the main treatments. Most of the studies found are case series without control groups and/or participants were selected under biased conditions or non-independently from evaluation researchers. Moreover, poor explanations about treatment dosage were given in most of the cases, with high heterogeneity among the trials: one measured the dosage in joules [21], another facilitated the frequency of emission in kilohertz [20], another referred to a power percentage in watts and, in others, temperature was measured at the tissue surface [28,29]. On this line, future studies must explain the dosage according to frequency of emission, the pulse of the signal (continuous or pulsed in a percentage of time), intensity and periodicity of treatment. Although tissue surface temperature could also be interesting to investigate, due to types of emission temperature differing significantly from surface to deeper tissues [41], the latter is the target of treatment in most cases.

Urinary incontinence was the most treated pelvic floor condition, obtaining favorable results in all the studies, both in women and prostatectomized men. The RCT of Leibaschoff et al. evidenced significant improvements after short RFD applications where temperature remained under 45 °C [29], which is the top temperature threshold within safety limits [42]. The rest of the studies were case series that performed similar applications regarding temperature increment [23,24,34], with changes in frequencies and place of the application with similar results, so it seems that the key point for effectiveness could be related with reaching a temperature between 40 and 45 °C for almost two minutes [23,24,29,34]. Only Razzaghi et al. extended the treatments to 3 weeks [31]. Prostatectomized men mentioned discomfort while introducing the endoanal applicator, this being the only side effect mentioned [34]. The lack of control groups in the case series reduced the possibility of reaching stronger conclusions, as there was a high risk of selection and comparison bias.

Pelvic floor pain was also a frequent outcome in the results of this review. Postpartum perineal pain and related disturbances in the first and second day seem to have poor improvements with RFD, although participants mentioned a reduction in painkillers intake, which is positive despite no changes in pain being observed [20]. More success was obtained by Fernández-Cuadros et al. with chronic pelvic pain and dyspareunia participants, observing an important reduction in pain and improvements in pelvic floor muscle strength [32]. However, the lack of a control group limits the strengths of this case series, as well as the heterogeneity of the participants selected; thus, a randomized clinical trial would be needed to confirm these results. Another condition where the effects of RFD were considered was endometriosis, a complex syndrome that severely affects women, conditioning their whole life [30]. Five participants were intensively treated over three menstrual cycles, obtaining improvements in dysmenorrhea intensity, sex interference and myofascial and referral pain [30]. These results support the design of a larger trial, as results cannot be generalizable due to sample limitations and inexistence of a control group. Last but not least, 96 participants with Peyronie’s disease benefit from RFD treatment, obtaining a reduction of 2 points in VAS during erection [27]. No changes were observed in penis curvature, despite RFD being accepted as an influence in collagenesis and fibroblast proliferation because of the heat produced in the tissue [15,43].

Two RCTs, a non-randomized trial and a case series assessed the effects of RFD in sexual function and orgasmic dysfunction. Both RCTs show significant improvements in FSFI after treatment [21,28]. Similar results were obtained by Alinsod et al. in a case series with 25 participants, although the changes were self-reported by the participants based on time to orgasm, which may lead to bias. Notwithstanding, the non-randomized trial showed no improvements in FSFI, which makes us think that the differences should be based on the treatment applied [22]. In this case, an intensive treatment regimen (daily [21] or weekly [28]) would be more effective than a monthly one [22], as temperatures reached in the treatments were similar.

This review also found RFD applications that targeted pelvic floor muscles contraction. Dayan et al. observed that the treatment with RFD produced an increment in maximal pelvic floor muscles contraction but no changes were perceived in base muscle tone [33]. Similar results regarding muscle strength were reported by Fernández-Cuadros et al. [35]. In any case, once again, these results must be considered with caution due to the low-quality evidence that the case series offer.

Concerning the safety of the RFD application in the pelvic floor muscles, five studies identified that the treatment presents a low risk of adverse effects due to their lack of appearance [22,23,24,31,33]. Only Krychman et al. reported some adverse events, which resolved spontaneously [21].

The limited data do not allow us to compare between different types of RFD application. Only one study used a monopolar dielectric transmission device [30], which is relevant because this system produces low surface heating [44] compared with the capacitive–resistive method. The rest of the studies used bipolar devices, although some of them were named as monopolar (despite being composed of a return plate and an applicator) or quadripolar (despite only having two poles: the emitting and the return poles). This issue is probably due to marketing concerns over technical characteristics and must be clarified in future works.

Findings of this systematic review must be taken into account with caution due to the presence of several limitations. (1) This review combines randomized controlled trials, observational studies and case series, which reduces the quality of the evidence. (2) The low number of studies included and the low number of participants reported can reduce the accuracy and generalization of our findings. (3) The data publication limits may have left out some studies, although it must be considered that this is a new application and that no studies were found before 2016. (4) The low-moderate methodological quality of the studies included increases the risk of bias. (5) It is important to consider the possible risk of publication bias in our finding that can affect to the true effect of experimental therapy. (6) The last limitation is related with the level of evidence of our findings. According to the Oxford Centre for Evidence-Based Medicine, our review presents a level of evidence of III, due to it being a review composed of different study designs, with the majority being non-RCTs. The findings of this review may be classified as level C of the recommendation, due to the scientific evidence being conflicting and the fact that new studies may change our findings. However, meta-analysis could not be performed because studies did not report homogenous quantitative data to be integrated. Further research must be carried out to increase knowledge of the effect of non-invasive radiofrequency diathermy in pelvic floor disorders, especially randomized clinical trials that include follow-up of the participants.

## 5. Conclusions

Despite having low-quality evidence, the main findings in our review showed that RFD could be effective for improving urinary incontinence, pelvic floor muscles’ strength, sexual function and different pelvic pain conditions. Nevertheless, the high risk of bias among the existing literature forces us to consider these results with caution. In addition, it is necessary to carry out more clinical trials to assess the effect of RFD on different pelvic floor outcomes, minimizing the risk of selection and confusion bias.

## Figures and Tables

**Table 1:** Bibliographic search strategy used in each database.

DATABASES	SEARCH STRATEGY
PubMed Medline	(radiofrequency therapy [mh] OR radiofrequency therapy [tiab] OR “radiofrequency” [tiab] OR hyperthermia, induced [mh] OR hyperthermia, induced [tiab] OR induced hyperthermia [tiab] OR diathermy [mh] OR diathermy [tiab] OR capacitive–resistive therapy [tiab] OR dielectric radiofrequency [tiab] OR dielectric radiofrequency therapy [tiab]) AND (pelvic floor [mh] or pelvic floor [tiab] OR pelvic floor disorders [mh] OR pelvic floor disorders [tiab] OR pelvic floor diseases [tiab] OR pelvic floor dysfunction [tiab] OR urogenital diseases [mh] OR urogenital diseases [tiab] OR female urogenital diseases [tiab])
SCOPUS	(TITLE-ABS-KEY ((“radiofrequency” OR “diathermy” OR “capacitive–resistive” OR “hyperthermia”)) AND TITLE-ABS-KEY ((“pelvic floor” OR “pelvic floor disorders” OR “pelvic floor diseases” OR “female urogenital diseases” OR “pelvic floor dysfunction”)))
Web of Science	TS = ((* radiofrequency * OR * diathermy * OR * capacitive–resistive * OR * dielectric * OR * hyperthermia *)) AND TS = ((* pelvic floor * OR * pelvic floor disorders * OR * pelvic floor diseases * OR * female urogenital diseases * OR * pelvic floor dysfunction *))

**Table 2:** Summary of the randomized controlled trials selected.

Author, Year	Study	Disorder	*n*	Outcomes	Intervention	Results	PEDro
Bretelle et al., 2020	RCT	Postpartum perineal pain	60 EG(29) CG(31)	Perineal Pain (VAS), discomfort while walking and sitting (yes/no), analgesic intake	EG: 15′ of RFD (range 300–500 KHz) 1st and 2nd day of postpartum; CG: the same but sham therapy	Improvements in discomfort while walking and analgesic Intake in favor of experimental group, not in perineal pain (VAS)	5/10
Krychman et al., 2017	RCT	Sexual function	186 EG(123) CG(63)	Sexual function (FSFI, FSDS-R)	EG: cooled RFD (90 J/cm^2^); CG: RFD (1 J/cm^2^)	Improvements of 1.8 points in FSFI (*p* = 0.031) and 2.42 in FSDS-R	6/10
Pavone et al., 2017	RCT	Peyronie’s disease	96 EG(64) CG(32)	Erectile function (IIEF-5), quality of life (SF-36), pain during erection (VAS), penis curvature (°)	EG: 3 sessions (in 3 days) 5–8′ CAP (45%W) and 3′ RES (40%W); CG: the same without energy transfer	2-point (VAS) reduction in pain during erection (*p* > 0.01), no changes in IIEF-5 or curvature	5/10
Lordelo et al., 2016	RCT	Sexual function	43 EG(21) CG(22)	Sexual function (FSFI)	EG: 8 sessions of RFD (7 days between session). RFD applied until tissue reached 39–41 °C, then 2′ more of treatment; CG: previously heated gel with no emission	3.51 points (FSFI) of improvement for experimental group (*p* > 0.03)	7/10
Leibaschoff et al., 2016	RCT	Menopausal Urinary Symptoms	20 EG(10) CG(10)	Stress urinary incontinence (UDI-6, ICQ-SF UI), dyspareunia and dryness (VAS); Vaginal Health (VHI)	EG: 3–5′ RFD 40–45°; CG: sham RFD (the same without any heat)	Improvements in ICQ-SF UI and UDI-6 (*p* < 0.01) for experimental group	5/10

Abbreviations: *n*: sample size; RFD: radiofrequency diathermy; RCT: randomized controlled trial; EG: experimental group; CG: control group; VAS: visual analogue scale; FSFI: Female Sexual Function Index; FSDS-R: Revised Female Sexual Distress Scale; °: goniometer degrees; IIEF-5: International Index of Erectile Function; SF-36: SF-36 Health Survey; UDI-6: Urogenital Distress Inventory; ICQ-SF UI: International Consultation on Incontinence Questionnaire—Urinary Incontinence Short Form; VHI: Vaginal Health Index; KHz: kilohertz; ′: minutes; CAP: capacitive; RES: resistive.

**Table 3:** Summary of the other design of studies considered of interest.

Author, Year	Study	Disorder	*n*	Outcomes	Intervention	Results	NOS
Fortún et al., 2022	Case series	Endometriosis-related pain	5	Sex interference (EHP), MTP, pain (VAS), allodynia, neuropathic pain component (DN4)	25 RFD sessions along 3 menstrual cycles, 30′ per session	Improvements in sex interference, dysmenorrhea intensity, myofascial and referral pain.	***
Razaghi et al. 2021	Case series	Stress urinary incontinence	28	Urinary incontinence (I-QOL, Q-tip test, 24 h pad test)	Once a week for 3 weeks, 10′ heating at 40 °C the vaginal wall (pulsed emission at 20–40 w and 1000–300 kHz)	Significant improvements in I-QOL score and the pad test	**
Fernández-Cuadros et al., 2020	Prospective study	Women chronic pelvic pain and dyspareunia	37	Pain (VAS), pelvic floor muscles strength (mmHg)	8 session of pelvic floor muscles therapeutic exercise and 15′ RFD (5′capacitive +10′resistive)	Improvements in pain (3.52 VAS points) and muscle strength (both *p* < 0.0001)	***
Dayan et al., 2019	Case series	Postpartum restoration	50	Pelvic muscle tone and maximal contraction (biofeedback device)	2 (*n* = 31) or 3 (*n* = 19) sessions of RFD	Improvement in maximal pelvic floor contraction, no changes in tone	***
Sodre et al., 2019	One-arm clinical trial	Men urinary incontinence after radical prostatectomy	10	Pelvic floor muscular strength unidigital introduction (OGS), urinary incontinence (ICIQ-SF and ICIQ-OAB)	Endoanal RFD at 1 MHz and 3–4 kilojoules, 41 °C temperature (once temperature was reached, application stayed for 2′)	Decrease in urinary loss (*n* = 9) and resolution in *n* = 3	***
Wilson et al., 2018	Non-randomized trial	Women stress urinary incontinence and orgasmic dysfunction	10	Sexual satisfaction (MSSQ and FSFI)	3 RFD at 4-week intervals, 30′ per session at a temperature of 42–45 °C	Non-significative Improvements in stress urinary incontinence nor orgasmic dysfunction	*
Caruth et al., 2018	Case series	Women urinary incontinence	30	Urinary incontinence (ICIQ-UI and IIQ), pelvic floor impact (PFIQ-7)	RFD in vaginal canal: Group 1 (16–20′), Group 2 (10–12′), Group 3 (6–8′), 43 °C maximum	Improvements for all outcomes at two months follow-up (*p* < 0.001)	*
Lordelo et al., 2017	Case series	Women stress urinary incontinence	10	Pelvic floor muscular strength unidigital introduction (OGS), urinary loss (pad test)	Urethral meatus RFD, once per week along 5 weeks, 39–41 °C temperature (once temperature was reached, application stayed for 2′)	General improvement (*p* = 0.028) in urinary loss: 70% show reduction, 20% resolve and 30% worsening (pad test)	***
Vicariotto et al., 2016	Case series	Premenopausal and postmenopausal symptoms	25	Urinary incontinence (PISQ-12)	Four 10′ sessions of RFD, one each 10 days	Improvements in self-perceived dysuria/urinary incontinence and sexual function	*
Alinsod et al., 2016	Case series	Women orgasmic dysfunction	25	Self-perceived time to orgasm	Three 25′ sessions of RFD in a month, elevating temperature between 40 and 45 °C on clitoris region	Reduction of 50% in time to orgasm	-

Abbreviations. *n*: sample size; NOS: Newcastle and Ottawa Scale; EHP: Endometriosis Health Profile; MTP: myofascial trigger points; VAS: Visual analogue scale; ICIQ-SF: International Consultation on Incontinence Questionnaire Form; PISQ-12: Urinary incontinence sexual questionnaire; ICIQ-OAB: International Consultation on Incontinence Questionnaire Overactive Bladder; ICIQ-UI: International Consultation on Incontinence Questionnaire—Urinary Incontinence; OGS: Oxford Grading Scale; MSSQ: Millheiser Sexual Satisfaction Questionnaire; FSFI: Female Sexual Function Index; PFIQ-7: short version of Pelvic Floor Impact Questionnaire; IIQ: short form of Incontinence Impact Questionnaire; *: number of stars obtained in NOS.

## Data Availability

The data of the study are available under request to the corresponding author.

## Supplementary Materials

The following supporting information can be downloaded at: https://www.mdpi.com/article/10.3390/medicina58030437/s1, Supplementary Table S1. PEDro Assessment Scores. Supplementary Table S2. Newcastle-Ottawa Assessment Scores.
