# Supplementary material for: Effects of Non-Invasive Radiofrequency Diathermy in Pelvic Floor Disorders: A Systematic Review
Source: Medicina (Kaunas). 2022 Mar 17;58(3):437. doi: 10.3390/medicina58030437 (PMC8951766; doi:10.3390/medicina58030437)
Supplement: Supplementary file 1 [file medicina-58-00437-s001.zip › medicina-1561256-supplementary.pdf]

SUPPLEMENTARY TABLES

Supplementary Table S1. PEDro Assessment Scores.

Items / Authorship	2	3	4	5	6	7	8	9	10	11	TOTAL
**Bretelle et al., 2020**	Yes	No	No	Yes	No	Yes	No	Yes	Yes	No	5
**Krychman et al., 2017**	Yes	Yes	No	Yes	No	No	Yes	Yes	Yes	No	6
**Pavone et al., 2017**	Yes	Yes	Yes	Yes	No	No	Yes	No	No	No	5
**Lordelo et al., 2016**	Yes	Yes	Yes	Yes	No	Yes	No	Yes	Yes	No	7
**Leibaschoff et al., 2016**	Yes	No	Yes	Yes	No	No	Yes	No	Yes	No	5

Abbreviations: 2 = Random allocation, 3 = Concealed allocation, 4 = Baseline comparability, 5 = Blind subjects, 6 = Blind therapists, 7 = Blind assessors = 8. Adequate follow-up, 9 = Intention-to-treat analysis, 10 = Between-group comparisons, 11 = Point estimates and variability.

Supplementary Table S2. Newcastle-Ottawa Assessment Scores.

Authorship	Selection				Comparability	Exposure			Total Score
	S1	S2	S3	S4	C1	E1	E2	E3	
**Fortún et al., 2022**	*	*				*			***
**Razaghi et al. 2021**	*					*			**
**Fernández-Cuadros et al., 2020**	*	*				*			***
**Dayan et al., 2019**	*	*				*			***
**Sodre et al., 2019**	*	*				*			***
**Wilson et al., 2018**						*			*
**Caruth et al., 2018**						*			*
**Lordelo et al., 2017**	*	*				*			***
**Vicariotto et al., 2016**						*			*
**Alinsod et al., 2016**									-

Abbreviations: S1 = Adequate case definition; S2 = Representativeness of the cases; S3 = Selection of controls; S4 = Definition of controls; C1 = Comparability of cases and controls; E1 = Ascertainment of Exposure; E2 = Same method of ascertainment for cases and controls; E3 = Non-Response rate; *: number of stars obtained in NOS.
